# Sensory Processing Difficulties in Children and Adolescents with Obsessive-Compulsive and Anxiety Disorders

**DOI:** 10.1007/s10802-022-00962-w

**Published:** 2022-09-23

**Authors:** Matti Cervin

**Affiliations:** Department of Clinical Sciences Lund, Lund University, Sofiavägen 2D, SE-22241 Lund, Sweden

**Keywords:** OCD, anxiety disorders, sensory processing, sensory sensitivity, children, adolescents

## Abstract

Altered sensory processing has been linked to symptoms of obsessive-compulsive disorder (OCD) and anxiety disorders (ADs) in youth, but few studies have examined sensory processing in clinical samples and no study has analyzed self-report data from youth meeting diagnostic criteria for OCD or ADs. This study included 86 youth with OCD, 82 youth with ADs, and 46 youth without psychiatric disorders. Participants completed the adolescent version of the Sensory Profile and scales measuring three symptom dimensions of OCD, four symptom dimensions of anxiety, and symptoms of major depression. Results showed that different forms of sensory processing difficulties (sensitivity, avoidance, low registration) were adequately captured by one broad sensory processing factor. Youth with OCD and ADs reported statistically significantly more sensory difficulties than youth without psychiatric disorders, but the two clinical groups did not differ from each other. Altered sensory processing in the clinical groups was not explained by the presence of neurodevelopmental disorders. Sensory difficulties were moderately to strongly related to all self-reported symptom dimensions, and uniquely related to the OCD dimension of symmetry/ordering and the anxiety dimensions of panic and social anxiety. Most youth in the clinical groups were classified as having difficulties with sensory processing. The present study shows that sensory processing difficulties are common in youth with OCD and ADs, not explained by co-occurring neurodevelopmental disorders, and linked to a host of internalizing symptoms. More research is needed to identify whether sensory processing difficulties precede, follow, or mutually reinforce the development of OCD and ADs in youth.

## Introduction

Obsessive-compulsive disorder (OCD) and anxiety disorders (ADs) are prevalent in childhood and adolescence with prevalence rates for OCD ranging from 1 to 4% (Geller, 2006) and ADs affecting as many as 30% of youth (Merikangas et al., 2010). Age of onset for OCD and AD is most common before adulthood (Solmi et al., 2021), making research with children and adolescents important. OCD and ADs are heterogeneous with respect to symptom expression, impairment, and course (Beesdo et al., 2009; Cervin et al., 2021), further complicating research aimed to identify factors involved in the development, exacerbation, and maintenance of the disorders.

There is a large research literature on how cognitive and emotional functioning may be involved in OCD and ADs, and cognitive and emotional components are well represented in modern research initiatives such as the Research Domain Criteria (RDoC) framework. RDoC aims to characterize healthy and disordered mental functioning along six domains: positive valence, negative valence, cognitive systems, social processes, arousal/regulatory systems, and sensorimotor systems (Harrison et al., 2019; Insel et al., 2010). Largely missing from prior work, and from the RDoC model of psychopathology, is the role of the sensory system.

Sensory perception is a prerequisite for human functioning and sensory input can be perceived through auditory, tactile, visual, olfactory, vestibular, gustatory, or proprioceptive processing. Although the same senses are typically triggered by the same type of stimuli across individuals, individual processing of sensory stimuli differ greatly. Some people react strongly while others barely react at all. This phenomena is explained by individual differences in sensory sensitivity, which has been described as the degree to which we react to changes in stimuli intensity (Schauder & Bennetto, 2016). Several factors may contribute to individual differences in sensory sensitivity, for example, general perceptual reactivity, gating (the ability to filter out stimuli), and habituation (how quickly the sensory reaction decreases when exposed to repeated stimuli). In a recent twin study, 47% of the variation in sensory sensitivity was explained by inherited genetic variants (Assary et al., 2021), suggesting that sensory sensitivity is a moderately heritable trait. This is in line with child studies showing that the association between sensory processing and psychiatric symptoms may be accounted for by common underlying genetic effects (Van Hulle et al., 2012). Further, research in non-human animals suggests that a range of traits can be traced back to variation in core sensitivity to the environment, making sensory sensitivity a potential important meta-trait governing other traits also in humans (Aron et al., 2012).

Sensory processing has high face validity in relation to mental disorders since many mental health symptoms are characterized by exaggerated reactions to external and internal stimuli and difficulties in downregulating these reactions. The literature on sensory processing difficulties in mental and neurodevelopmental disorders in youth is small, but most studies have found that sensory difficulties are linked to these disorders (Kotsiris et al., 2020). The majority of studies have been conducted in relation to autism spectrum disorder (ASD), showing that altered sensory processing is associated with poorer adaptative functioning and attention difficulties in youth with ASD (Dellapiazza et al., 2018; Kotsiris et al., 2020). The emphasis on ASD is not surprising given that sensory processing is included in the diagnostic description of ASD in the DSM-5 (American Psychiatric Association, 2013).

The few studies that have examined sensory processing and internalizing symptoms in children and adolescents strongly support a link between the two. In a seminal study, Ben-Sasson et al., (2009) showed that in representative sample of children (aged 7–11 years), parent-rated sensory over-responsivity was associated with a range of child mental health problems, with the strongest associations emerging in relation to internalizing symptoms. In a recent study, Burgard et al., (2022) found that self-reported sensory sensitivity in youth aged 9–18 years was significantly and moderately correlated with depression and anxiety.

Only two studies have examined sensory processing in clinical samples of youth with diagnosed internalizing disorders. In a study with 88 children and adolescents (aged 4–17 years) who seeked care for anxiety or OCD, parents reported on sensory processing in their children using the Sensory Over-Responsivity Inventory (Conelea et al., 2014). A large majority (93%) of children were reported to be bothered by at least one sensory difficulty (e.g., tactile, sound) and parent-reported sensory difficulties were associated with OCD symptoms, depression, and functional impairment. In a study with 80 children and adolescents with OCD (mean age = 8.9 years) (Lewin et al., 2015), the authors analyzed parent-reported sensory processing using the Short Sensory Profile. The authors found that sensory hypersensitivity was associated with OCD symptoms and impairment, but that the association with impairment was moderated by OCD severity. Further, the OCD symptom dimensions of contamination/cleaning and symmetry/ordering were significantly associated with sensory difficulties.

These two studies indicate that youth seeking care for OCD and/or ADs may present with clear sensory processing difficulties, but the literature is small and has limitations. First, no study has compared different groups of clinical children (e.g., different disorders), which results in lack of clarity about whether sensory processing difficulties are more pronounced in some internalizing disorders. Second, no study has examined how sensory processing is related to symptoms across the major internalizing symptom dimensions in youth (e.g., depression, different types of OCD, social anxiety, panic). This is important as internalizing symptoms in youth are dimensional rather than binary (Kotov et al., 2017). Third, no clinical study has used a youth-reported sensory processing scale, which is a concern since sensory processing is a highly internal phenomena and though may be of limited access to others (e.g., parents). Fourth, despite the potential link between sensory processing and OCD, there is very little work on associations between sensory processing and the major symptom dimensions of OCD (i.e., disturbing thoughts/checking, contamination/cleaning, and symmetry/ordering).

The aim of this study is to address the above limitations. Self-reported sensory processing data from youth with a principal OCD or AD diagnosis was analyzed and compared to data from youth without psychiatric disorders. Group differences were examined and dimensional associations between sensory processing and internalizing symptom dimensions estimated. It was expected that the clinical groups would report more difficulties with sensory processing than youth without psychiatric disorders. It was also expected that sensory processing difficulties would be most clearly associated with OCD, particularly contamination/cleaning and symmetry/ordering symptoms.

## Methods

### Participants

Participants were 213 youth (aged 7–18 years) of which a majority (70%) were girls. Sociodemographic and clinical characteristics of the three samples (OCD, ADs, non-clinical) are in Table 1. All clinical participants were seen at a child and adolescent mental health unit in the south of Sweden between 2015 and 2019. The non-clinical comparison group was recruited from local schools during the same period. The principal disorders in the AD group were generalized anxiety disorder (39%), social anxiety disorder (33%), panic disorder (11%), specific phobia (11%), and separation anxiety disorder (6%). In the OCD group, 55.3% had a comorbid anxiety disorder and the most common comorbid disorder was generalized anxiety disorder (34.1%).

**Table 1:** Sociodemographic information, clinical characteristics, and scores on study measures. Group differences were examined using one-way ANOVAs (with follow-up Tukey-corrected tests), chi-square tests, and Fisher’s exact tests

		OCD *n* = 85	Anxiety Disorders *n* = 82	Non-clinical *n* = 46	Group differences
**Sociodemographic information**					
	Age, *M* (SD)	13.77 (2.53)	14.82 (2.48)	14.21 (3.05)	OCD < Anxiety = Non-clinical
	Female, *n* (%)	52 (61%)	68 (83%)	30 (65%)	Anxiety > OCD = Non-clinical
	Living with both parents, *n* (%)	54 (64%)	45 (55%)	26 (57%)	No significant differences
	Family economy, good or better, *n* (%)	64 (80%)	42 (70%)	30 (81%)	No significant differences
	Mother with university education, *n* (%)	66 (79%)	38 (46%)	34 (89%)	Anxiety < OCD = Non-clinical
	Father with university education, *n* (%)	43 (51%)	38 (56%)	28 (78%)	Non-clinical > Anxiety = OCD
**Diagnostic information**					
	Major depression, *n* (%)	10 (12%)	26 (32%)	0 (0%)	Anxiety > OCD > Non-clinical
	Anxiety disorder, *n* (%)	47 (55.3%)	82 (100%)	0 (0%)	Anxiety > OCD > Non-clinical
	OCD, *n* (%)	85 (100%)	0 (0%)	0 (0%)	OCD > Anxiety = Non-clinical
	Generalized anxiety disorder, *n* (%)	29 (34.1%)	43 (52.4%)	0 (0%)	Anxiety > OCD > Non-clinical
	Neurodevelopmental disorder, *n* (%)	20 (24%)	11 (14%)	0 (0%)	Non-clinical < OCD = Anxiety
	Attention-deficit/hyperactivity disorder, *n* (%)	17 (20.0%)	10 (12.8%)	0 (0%)	Non-clinical < OCD = Anxiety
	Autism spectrum disorder	5 (5.9%)	2 (2.4%)	0 (0%)	No significant differences
**Sensory Profile**					
	Low registration, *M* (*SD*)	34.14 (8.18)	34.36 (8.82)	30.39 (8.27)	Non-clinical < OCD = Anxiety
	Sensation seeking, *M* (*SD*)	40.54 (7.11)	39.17 (7.91)	41.26 (8.19)	No significant differences
	Sensory sensitivity, *M* (*SD*)	35.83 (11.69)	38.50 (10.03)	29.21 (8.68)	Non-clinical < OCD = Anxiety
	Sensation avoiding, *M* (*SD*)	39.63 (11.10)	39.85 (9.25)	33.14 (9.17)	Non-clinical < OCD = Anxiety
**Symptom measures**					
	Depression, CDI-S (0–20)	5.61 (4.41)	7.87 (4.60)	2.23 (2.52)	Anxiety > OCD > Non-clinical
	Social anxiety, SCARED-R (0–14)	5.68 (3.83)	8.16 (4.33)	4.20 (3.45)	Anxiety > OCD = Non-clinical
	Panic anxiety, SCARED-R (0–26)	7.73 (5.99)	11.04 (5.77)	3.42 (3.17)	Anxiety > OCD > Non-clinical
	Generalized anxiety, SCARED-R (0–18)	8.31 (4.37)	10.46 (4.12)	4.83 (4.22)	Anxiety > OCD > Non-clinical
	Separation anxiety, SCARED-R (0–16)	5.37 (3.49)	5.70 (3.48)	2.52 (2.75)	Non-clinical < OCD = Anxiety
	Obsessing, OCI-CV (0–8)	4.44 (2.12)	3.61 (1.97)	1.55 (1.33)	OCD > Anxiety > Non-clinical
	Washing, OCI-CV (0–6)	3.09 (2.14)	1.33 (1.74)	0.77 (0.99)	OCD > Anxiety = Non-clinical
	Ordering, OCI-CV (0–6)	3.02 (2.11)	2.06 (1.71)	1.56 (1.78)	OCD > Anxiety = Non-clinical

## Measures

**Sensory Profile.** To measure sensory processing in everyday life, the Adolescent/Adult version of the Sensory Profile (Dunn & Brown, 2002) was used. It is a 60-item measure where respondents are asked to report how often they experience different sensory situations/experiences. Items are rated on a 5-point Likert scale ranging from “Almost Never” to “Almost Always”. The measure is based on Dunn’s model where individual sensory processing depends on the interaction between (i) neurological sensory thresholds (i.e., the amount of stimuli needed for the nervous system to register these stimuli) and (ii) individual behavioral responses when thresholds are reached (Dunn, 1997). High and low reactivity paired with behavioral responses in accordance with or to counteract the activation (passive/active) yields four quadrants, which are also the basis for the proposed factor structure of the measure: low registration, sensation seeking, sensory sensitivity, and sensation avoiding. The items of the measure include different situations and different senses. Prior evaluations of the measure have supported adequate internal consistency and convergent and discriminant validity (Dunn & Brown, 2002). However, the internal consistency coefficients have been in the lower range and very few confirmatory factor analyses have been conducted to test whether the model/data fit of the proposed 4-factor model is adequate. Therefore, in this study, the psychometric properties of the measure were evaluated as part of the statistical analyses and results are presented in the results section. It is stated that the measure is most appropriate from 11 years of age (Dunn & Brown, 2002). In the present study, 29 participants (13.7%) were under 11 years of age, but only 14 participants (6.6%) were under 10 years of age. To secure adequate reporting, it was confirmed that all younger children had understood the item content. Further, there were only negligible differences in missing data (under 11 years of age = 1.5%; 11–17 years of age: 2.3%) and internal consistency (alpha for the full measure was 0.91 in those under 11 years of age and 0.90 in the 11–17-year-olds), indicating that the measure worked similarly across age groups.

**Symptom measures.** The Obsessive Compulsive Inventory – Child Version (OCI-CV) was used as a self-report measure of OCD symptom dimensions (Foa et al., 2010). The three scales that best represent the three major symptom dimensions of OCD were used: the OCI-CV scale of washing as an indicator of contamination/cleaning, the OCI-CV scale of obsessing as an indicator of disturbing thoughts/checking, and the OCI-CV scale of ordering as an indicator of symmetry/ordering (Cervin, Garcia-Delgar, et al., 2022). The Screen for Child Anxiety Related Disorders (SCARED) was used to assess self-reported symptom dimensions of anxiety (social anxiety, panic, generalized anxiety, and separation anxiety) (Birmaher et al., 1997). The 10-item version of the Children’s Depression Inventory (CDI-S) was used to assess self-reported depressive symptoms (Allgaier et al., 2012). Psychometric properties of the scales were evaluated as part of the statistical analyses and results are presented in the results section.

**Children’s Yale-Brown Obsessive Compulsive Scale (CY-BOCS).** The severity of OCD was assessed using the interview-rated CY-BOCS. This is a 10-item scale that assesses the frequency, interference, distress, resistance, and control for obsessions and compulsions, respectively (Scahill et al., 1997). It yields a score of 0–40 with higher scores indicating more severe OCD symptoms. A cut-off score equal to or above 14 on the CY-BOCS has been shown to best indicate clinical severity of pediatric OCD and scores equal to or above 22 indicate moderately severe OCD (Cervin, OCD Severity Benchmark Consortium, et al., 2022). The internal consistency of the items of the CY-BOCS was adequate using the present sample (*a* = 0.75). According to the international severity guidelines (Cervin, OCD Severity Benchmark Consortium, et al., 2022), the scores on CY-BOCS indicated that 37% of the OCD sample had mild OCD, 58% moderately severe OCD, and 5% severe OCD.

**Mini-International Neuropsychiatric Interview for Children and Adolescents (MINI-KID).** The diagnostic status of all participants, including those in the comparison group, was assessed using the structured diagnostic interview, the MINI-KID (Sheehan et al., 2010). In this interview the most common youth psychiatric disorders are assessed. DSM-5 criteria were used for OCD and ADs. Diagnostic reliability is not available given that the interviews were conducted within a clinical practice. However, group comparisons on study measures showed clear differences in the expected direction, that is, the OCD group scored higher on OCD measures and the AD group higher on anxiety measures (see Table 1).

## Procedure

Treatment-seeking youth meeting criteria for OCD and/or an AD were invited to participate in the study. Inclusion criteria were (i) being 7–17 years of age and (ii) having either OCD or AD as a principal mental disorder. Exclusion criteria were (i) selective mutism or an anxiety disorder induced by substances, medication, or a medical condition, (ii) established or suspected intellectual disability in the moderate to severe range, (iii) another mental disorder and/or social problems that were in more immediate need of treatment than OCD or an AD, and (iv) not being fluent in Swedish. All clinical and non-clinical participants and their guardians provided written informed consent/assent and then proceeded to complete a diagnostic interview (conducted by a clinical psychologist) and study questionnaires (either at home or in the clinic). All clinical psychologists were trained in using the MINI-KID and received supervision. During the clinical interview, priorly confirmed diagnoses of attention-deficit/hyperactivity disorder (ADHD) and autism spectrum disorder (ASD) were recorded and used to indicate the presence of a neurodevelopmental disorder (see Table 1 for frequencies). During the interview, parents provided information about maternal and paternal education level, family living arrangements, and the economic situation of the child that was participating in the study (see Table 1). The study was approved by the regional ethics committee at Lund University, Sweden (Dnr2015/663-3/12 and Dnr2016/92 − 12/5).

### Statistical Analysis

It was first examined whether the proposed four-factor structure of the Sensory Profile (sensitivity, avoidance, low registration, sensation seeking; 15 items each) showed adequate model/data fit using this sample. The full sample was used to maximize statistical power and variation in responses. Confirmatory factor analysis (CFA) based on diagonally weighted least squares estimation because of the ordinal nature of the items was used. Model/data fit was evaluated by inspecting four fit indexes: CFI and TLI (values > 0.90 are indicative of adequate fit) and RMSEA and SRMR (values < 0.06 and 0.08, respectively, are indicative of good fit) (Schermelleh-Engel et al., 2003). Internal consistency was evaluated using Chronbach’s alpha and McDonald’s omega with values above 0.70 being considered adequate.

Group differences in sensory processing were examined within a structural equation modeling (SEM) environment, where the sensory variables were regressed onto group factors (e.g., OCD vs. ADs) while accounting for differences in age and sex. Associations between sensory variables and symptom dimensions were also estimated within a SEM environment by (i) letting the variables correlate freely and (ii) regressing the sensory variables onto the symptom dimensions. When examining associations between sensory processing and symptom dimensions all participants were pooled to increase variation and get better representation of observations across the dimensionality of both sensory processing and symptom dimensions. This approach is recommended when examining links between core mental processes and symptoms of mental disorders in the RDoC initiative (Cuthbert, 2014).

Participants were also classified according to the degree of difficulties they reported within each sensory quadrant. For this classification, the adolescent norms from the Sensory Profile manual were used and the classification procedure included in the manual was followed, where participants > 1 SD above the mean were classified as experiencing sensory difficulties within that sensory quadrant.

Missing data were present for living arrangements (OCD group: 0%, AD group: 13%, comparison group: 7%), family economy (OCD group: 6%, AD group: 27%, comparison group: 20%), maternal education level (OCD group: 1%, AD group: 17%, comparison group: 17%), and paternal education level (OCD group: 6%, AD group: 27%, comparison group: 22%). Missing data were also present on the item-level for all study measures but missingness was very low (Sensory Profile: 2.2%, OCI-CV: 0.5%, SCARED: 1.3%, CDI-S: 0.9%) and was handled by pairwise deletion in the statistical models. Missing demographic data were not handled since none of the variables with missingness were included in statistical analyses.

## Results

### Structure of Sensory Processing

Scores on study measures for the samples (OCD, ADs, non-clinical) are in Table 1. The model/data fit of the proposed factor structure of the Sensory Profile was poor (RMSEA = 0.05, CFI = 0.86, TLI = 0.86, SRMR = 0.09). The estimated parameters were inspected, and it was clear that all items of the Sensation seeking factor loaded very weakly (or not at all) onto this factor. Further, four items on the Low registration factor (items 23, 36, 39 & 41) had very weak standardized loadings (< 0.30). A factor model that excluded the Sensation seeking factor and the four items with weak loadings onto the Low registration factor exhibited good model/data fit (RMSEA = 0.05, CFI = 0.93, TLI = 0.93, SRMR = 0.08) and adequate internal consistency for all three factors (low registration, alpha = 0.85, omega = 0.83; sensory sensitivity, alpha = 0.86, omega = 0.85; sensation avoiding, alpha = 0.89, omega = 0.87). The factors correlated very strongly (standardized covariance coefficients: 0.74–0.93), indicating that they could be considered indicators of a broader sensory difficulty factor. This broad second-order factor was included in the model by using the three first-order factors as its indicators and a majority of subsequent analyses were conducted in relation to this broad factor.

## Group Differences in Sensory Processing

All group comparisons were conducted while accounting for differences in age and sex. The OCD and AD groups did not differ statistically significantly on the broad sensory processing factor (β = 0.02 [-0.14, 0.18], p = .83). The OCD group reported significantly more difficulties on the broad sensory processing factor compared to the non-clinical group and the estimated difference corresponded to a moderate effect size (β = 0.35 [0.21, 0.50], p < .001). When neurodevelopmental status (having ADHD or ASD) was included as an independent variable, having OCD was still associated with more difficulties with sensory processing (β = 0.31 [0.15, 0.47], p < .001). The AD group also scored significantly higher than the non-clinical group on the broad sensory processing factor, with the estimated difference corresponding to a moderate effect size (β = 0.34 [0.18, 0.50], p < .001). When neurodevelopmental status was included as an independent variable, having an AD was still associated with more difficulties with sensory processing (β = 0.31 [0.14, 0.47], p < .001).

Group differences on the three psychometrically valid first-order sensory factors (low registration, sensory sensitivity, and sensation avoidance) were also examined. The OCD and AD groups did not differ significantly on any factor (all *p*s > 0.41). The OCD group reported significantly more difficulties on all factors than the non-clinical group, low registration (β = 0.29 [0.11, 0.46], p < .01), sensory sensitivity (β = 0.37 [0.22, 0.52], p < .001), sensation avoidance (β = 0.34 [0.18, 0.50], p < .001). The AD group also reported significantly more difficulties on all factors than the non-clinical group, low registration (β = 0.24 [0.06, 0.41], p < .01), sensory sensitivity (β = 0.32 [0.15, 0.48], p < .001), sensation avoidance (β = 0.39 [0.24, 0.55], p < .001).

In the above analyses, a clear effect of both age and sex on sensory processing emerged. To estimate these effects within the clinical sample, a post hoc model with only clinical participants was conducted in which the sensory factor was regressed onto age and sex. Results showed that girls reported more sensory processing difficulties than boys (β = 0.20 [0.04, 0.35], p = .01) and that being older was positively associated with more sensory difficulties (β = 0.21 [0.05, 0.37], p = .01). Both effects were in the small to moderate range.

## Associations Between Sensory Processing and Symptom Dimensions

**Fig. 1:**
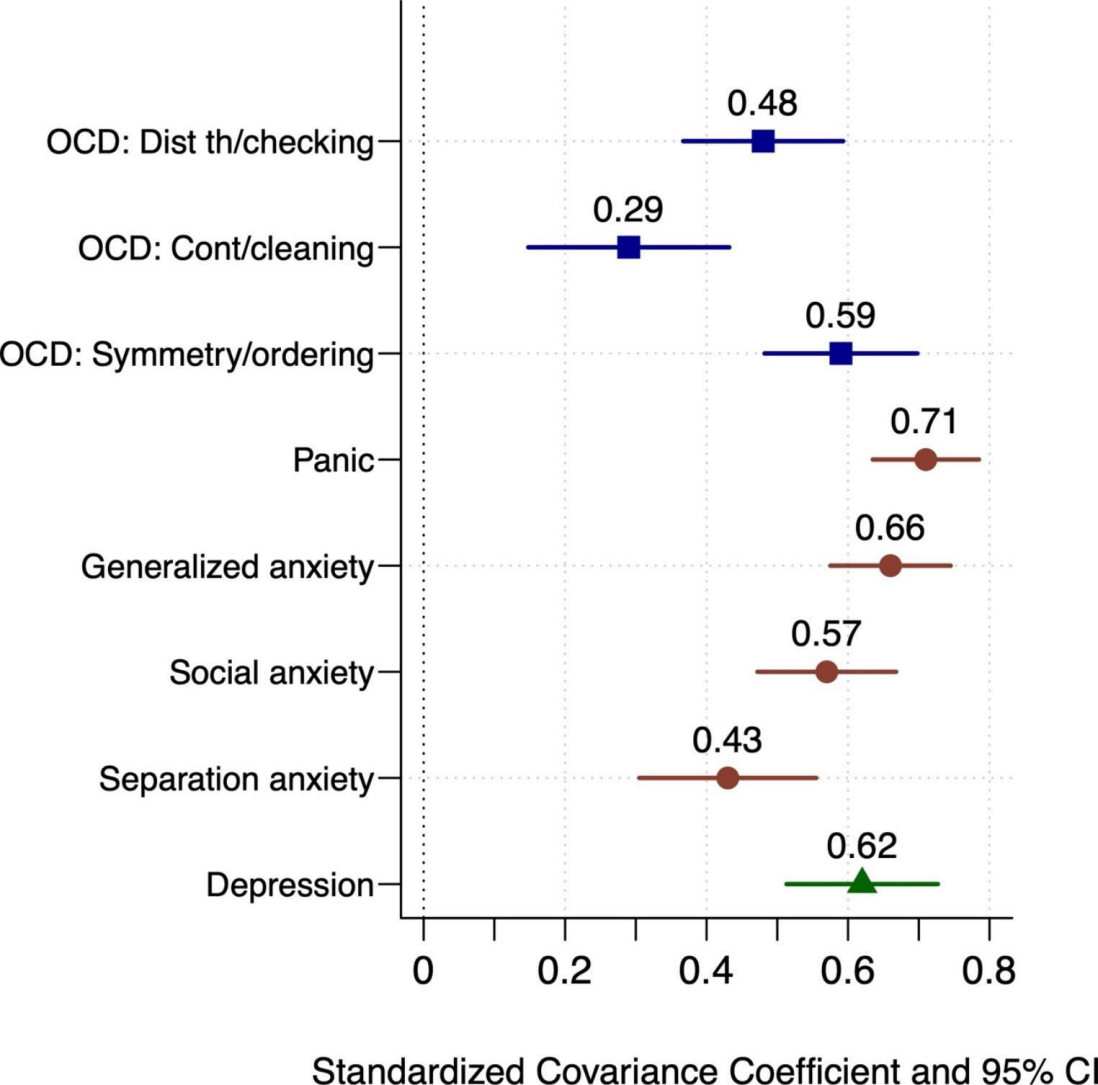
Standardized covariance coefficients between sensory processing difficulties and internalized symptom dimensions

The model/data fit of the model that included all symptom dimensions was adequate (RMSEA = 0.03, CFI = 0.92, TLI = 0.91, SRMR = 0.10) and all factors had adequate internal consistency (all *as* > 0.85). The broad sensory factor was significantly associated with all the measured symptom dimensions. The standardized covariance (correlation) coefficients between sensory processing and each of the symptom dimensions are presented in Fig. 1. To identify statistically unique associations, the sensory factor was regressed onto all the symptom dimensions, age, and sex. Three symptom dimensions were statistically significantly associated with the sensory factor: symmetry/ordering (β = 0.36, *p* < .001), panic (β = 0.40, *p* < .001), and social anxiety (β = 0.27, *p* < .001).

## Frequency of Sensory Difficulties

**Fig. 2:**
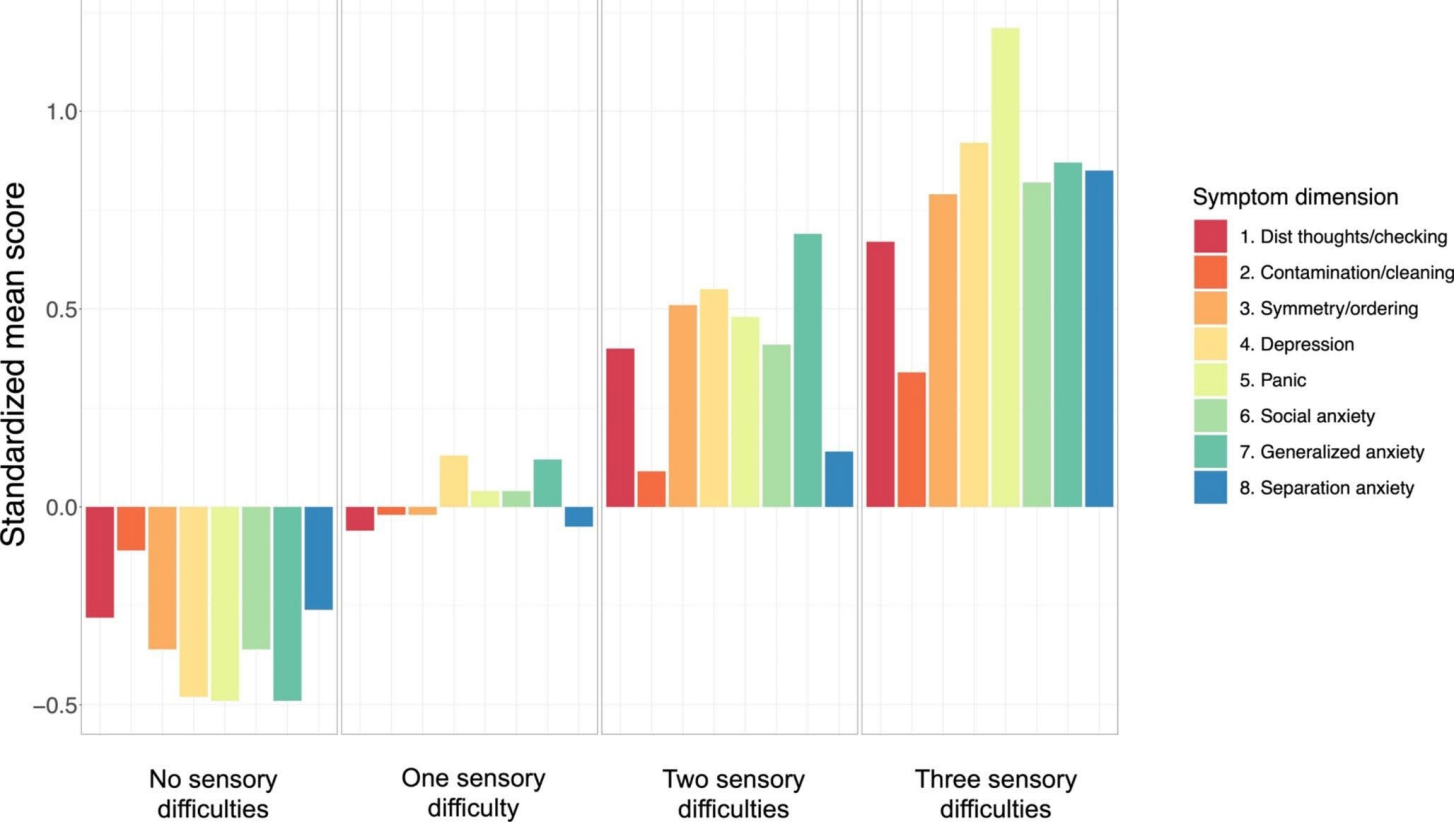
Standardized mean scores on the internalized symptom dimensions across participants with no, one, two, and three sensory difficulties

Participants were classified using the adolescent norms provided in the Sensory Profile manual. Classification was only performed for the three sensory factors/quadrants that showed adequate psychometric properties; thus, each participant could be classified as having 0–3 sensory difficulties. In the OCD group, 52% had zero sensory difficulties, 20% had one difficulty, 11% had two difficulties, and 18% had three difficulties. The proportions in the AD group were 40% – 22% – 21% – 17% and in the non-clinical group 67% – 20% – 11% – 2%. An ordinal regression model, accounting for age and sex, showed that the OCD group had more difficulties than the non-clinical group (OR = 2.78 [1.27, 6.05], *p* = .01). The association was somewhat attenuated and borderline significant when neurodevelopmental status was accounted for (OR = 2.23 [1.02, 5.03], *p* = .05). The AD group also had significantly more sensory difficulties than the non-clinical group (OR = 2.94 [1.36, 6.36], *p* < .01). The association was somewhat attenuated but still significant when neurodevelopmental status was accounted for (OR = 2.27 [1.03, 5.00], *p* = .04). No difference emerged when comparing the OCD and AD groups (*p* = .97). In Fig. 2 are standardized mean scores on the internalizing symptom dimensions (*M* = 0, *SD* = 1) across participants with 0, 1, 2, and 3 sensory difficulties. The figure shows that symptom severity increases for all symptom dimensions with each additional sensory difficulty.

## Discussion

The present study was carried out to examine the role of sensory processing in pediatric OCD and ADs. The expectation was that sensory processing difficulties would be most elevated in those with OCD and most clearly associated with OCD symptoms. This was not supported by data. Instead, sensory processing difficulties emerged as a ubiquitous characteristic of both pediatric OCD and ADs, and several results supported this conclusion.

First, youth with OCD and ADs reported substantially more difficulties with sensory processing than youth without psychiatric disorders and these differences were in the moderate range, with no significant difference between the two clinical groups. Second, when extending the analysis to dimensional severity across a range of internalizing symptom dimensions, sensory processing difficulties were related to all dimensions, which covered a broad range of diverse symptom expressions. Third, by using the norm scores provided by the Sensory Profile manual, it was clear that youth with OCD and ADs reported significantly more sensory difficulties than non-clinical youth, and there was a clear pattern showing that severity of symptoms across all symptom dimensions increased with each additional difficulty. Thus, all analyses supported that sensory processing difficulties are closely linked to OCD and ADs in youth.

Based on previous literature and the clear sensory involvement in OCD symptoms revolving around contamination/cleaning and symmetry/ordering (Cervin et al., 2021), it was expected that sensory processing difficulties would be most clearly associated with OCD. No strong support was found for this hypothesis since the OCD group did not differ significantly from the AD group on any sensory variable and sensory processing was associated with all assessed symptom dimensions. When unique associations between sensory processing and symptom dimensions were estimated, symmetry/ordering, panic, and social anxiety were the only symptom dimensions that were uniquely associated with sensory processing difficulties. Of note, only one of these three dimensions pertained to OCD symptoms (symmetry/ordering). Further, the presence of neurodevelopmental disorders (ADHD, ASD) did not explain the elevated sensory processing difficulties in youth with OCD or ADs, suggesting that sensory processing difficulties are broadly related to internalizing mental health symptoms in youth and not explained by co-occurring ASD, where sensory processing is included in the diagnostic criteria.

Two prior studies on sensory processing in clinical samples of youth with OCD/ADs have been published (Conelea et al., 2014; Lewin et al., 2015), with both studies indicating sensory difficulties in these disorders. The present study supports these findings but adds several important results. First, in contrast with the two previous studies, a non-clinical comparison group was included, and for the first time it was shown that youth with OCD or ADs have elevated sensory difficulties compared to non-clinical youth. Second, this is the first comparison of clinical groups of youth with internalizing disorders, finding no evidence for substantial differences in sensory processing between pediatric OCD and pediatric ADs. Third, in the present study, neurodevelopmental status was accounted for, showing that sensory difficulties in youth with OCD or ADs cannot be explained by the presence of for example ASD. Fourth, this study is the first examination of self-reported sensory difficulties in youth with OCD or ADs, with this being an incremental addition to the two prior studies that were based on parent-report.

Similarly to Conelea et al., (2014), sensory difficulties in this study were related to many different types of internalizing symptoms. Combined with negligible differences between the clinical groups and that no specific sensory domain stood out as especially difficult, the present results support that sensory processing difficulties in pediatric OCD and ADs are broad and non-specific. It is possible that such difficulties reflect underlying difficulties with self-regulation (Buckner et al., 2003), which refers to the capacity to control attention, cognition, emotion and behavior to support short- and long-term goals (Karoly, 1993).

Clinically, the results of the present study suggest that youth with clear sensory processing difficulties are of increased risk of a multitude of internalizing symptoms, justifying broad diagnostic assessments. Further, sensory processing difficulties should not be considered a sole indicator of ASD or other neurodevelopmental disorders, rather, such difficulties appear to be a transdiagnostic factor in relation to mental disorders in youth.

Among clinical participants, sensory processing difficulties were more common in girls and in older participants. Little work has examined sociodemographic and developmental differences in sensory processing difficulties in youth, but a previous study that included youth with OCD found that sensory over responsivity was more common among younger children (Lewin et al., 2015). In this previous study, parents completed the sensory measure, which may explain the conflicting findings. In adults, women generally have higher levels of sensory sensitivity than men (Dixon et al., 2016). It is worth noting that the age and gender differences in the present study largely mirror major epidemiological patterns in the development of internalizing symptoms during childhood and adolescence. First, girls report more difficulties with internalizing symptoms than boys and this difference emerges primarily during adolescence (Alloy et al., 2016). Second, there is a general increase in symptom severity and comorbidity during adolescence (Merikangas et al., 2010), which mirrors the present finding that older participants had more sensory difficulties. The identified age and gender differences in the present study alongside clear associations between sensory processing and internalizing symptoms suggest that measures of sensory processing can strengthen longitudinal investigations of the development of internalizing symptoms in youth.

Strengths of the study are two well-defined clinical samples of youth alongside a well-defined non-clinical sample, the use of a self-report measure of sensory processing, and continuous/dimensional scores across several internalizing symptom dimensions, but several limitations merit mentioning. First, the cross-sectional nature of the study precludes casual conclusions. Longitudinal studies that simultaneously measure sensory processing and symptoms of OCD, anxiety, and depression are needed to better understand the causal and reciprocal relations that may underlie these associations. Second, a well-established self-report scale of sensory processing was used, but it would have strengthened the study to combine this scale with behavioral and parent-reported measures to increase the precision and depth of the sensory processing data. Third, the samples were relatively small, and no formal power analysis was conducted. However, the precision of estimates was high and the lower part of the confidence interval for each association was far above zero, indicating that false positives are unlikely.

Sensory processing difficulties are frequent in youth with OCD and ADs and associated with a broad range of internalizing symptoms. The current findings strongly imply that sensory processing may play an important role in pediatric internalizing disorders, but the cross-sectional design precludes conclusions about whether sensory processing difficulties precede, follow, or mutually reinforce the development of OCD and ADs in youth.
